# The use of ^11^carbon methionine positron emission tomography (PET) imaging to enhance radiotherapy planning in the treatment of a giant, invasive pituitary adenoma

**DOI:** 10.1259/bjrcr.20160098

**Published:** 2017-01-09

**Authors:** Nicolette Taku, Olympia Koulouri, Daniel Scoffings, Mark Gurnell, Neil Burnet

**Affiliations:** ^1^Department of Oncology, University of Cambridge, Cambridge Biomedical Campus, Addenbrooke's Hospital, Cambridge, UK; ^2^Metabolic Research Laboratories, Wellcome Trust-MRC Institute of Metabolic Science, University of Cambridge, Cambridge, UK; ^3^National Institute for Health Research, Cambridge Biomedical Research Campus, Addenbrooke’s Hospital, Cambridge, UK; ^4^Department of Radiology, Addenbrooke's Hospital, Cambridge, UK

## Abstract

A 54-year-old male presented with visual loss owing to a giant, infiltrative pituitary adenoma. Following decompressive trans-sphenoidal surgery, the patient was referred for adjuvant radiotherapy. We describe the potential utility of ^11^carbon methionine positron emission tomography imaging in confirming the true extent of tumour infiltration, which included the cavernous sinuses and the bones of the skull base. The co-registration of positron emission tomography imaging to planning MR and CT imaging provided assurance of complete radiotherapy coverage of the target volume and assisted with the minimisation of collateral radiation dose to adjacent organs at risk.

Giant pituitary adenomas (GPAs) are defined as pituitary tumours ≥ 4 cm in maximum diameter. Although typically benign, they can present specific challenges for disease control.^1^ While neurosurgery remains the primary treatment modality (particularly when vision is compromised or threatened), a significant proportion of patients receive adjuvant radiotherapy-reflecting the difficulty in achieving gross total resection.^2,3^ Here, we describe a case in which ^11^carbon methionine positron emission tomography (PET) imaging was used in radiotherapy treatment planning to confirm the true extent of residual tumour following surgery, in a patient who was found to have a giant, infiltrative gonadotroph adenoma.

## Clinical presentation

A 54-year-old male presented with recent onset of worsening vision that had led him to stop driving. He had a history of left-sided amblyopia secondary to a congenital cataract and had suffered a left retinal detachment at the age of 37 years. He was otherwise well and was on no regular medication. Ophthalmic examination revealed a right temporal field defect to confrontation but with preserved visual acuity. He could only detect hand movements with the left eye. Physical examination was otherwise unremarkable and there were no clinical stigmata of endocrine dysfunction.

## Investigation/imaging findings

**Figure 1.**
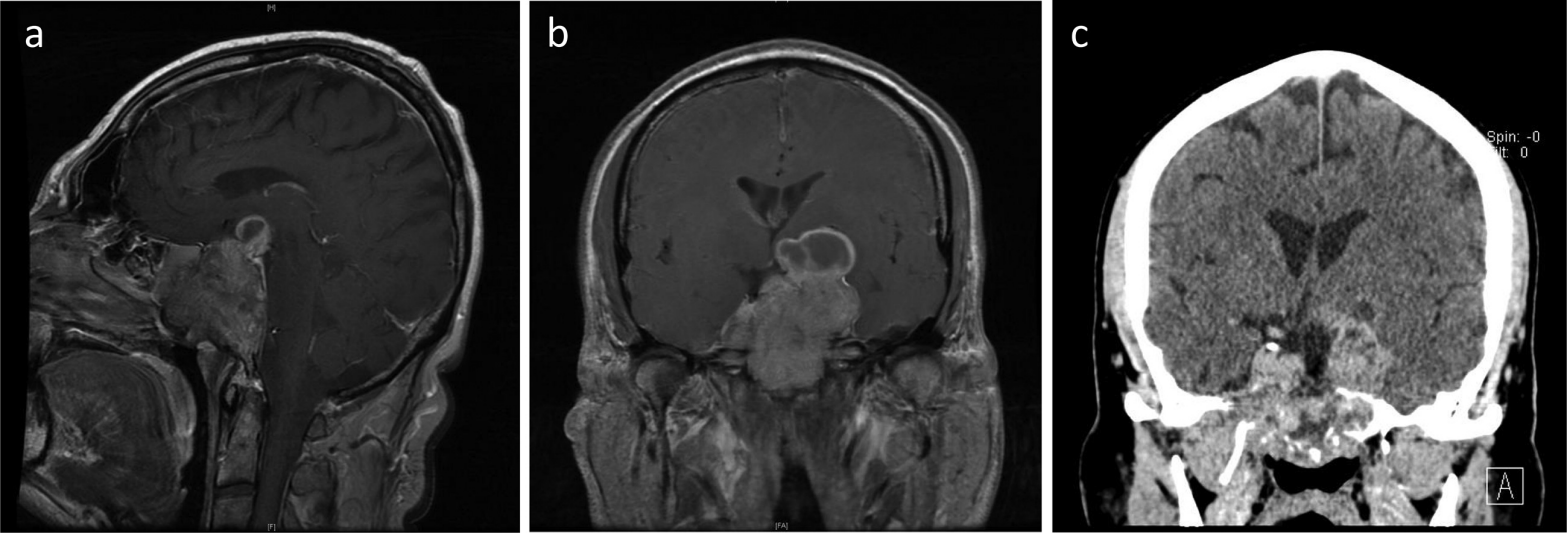
(a) Preoperative *T*_1_ weighted sagittal and (b) coronal MRI scan with contrast, and (c) CT with contrast demonstrated the large pituitary tumour with suprasellar extension, inferior invasion and destruction of the skull base.

An MRI scan of the pituitary demonstrated a large sellar mass (7 cm in maximum diameter), compressing the optic chiasm superiorly and extending inferiorly to the sphenoid sinus (Figures 1a,b). Additionally, a CT scan confirmed erosion of the skull base (Figure 1c). Endocrine profiling revealed mild hyperprolactinaemia, consistent with pituitary stalk disconnection syndrome [serum prolactin 430 mU l^–1^; reference range (RR) 45–375] and a raised serum follicle stimulating hormone (FSH) [63  U l^–1^; (RR 1.0–10.1)]. The remaining pituitary function tests were unremarkable. Ultrasound scanning of the testes excluded macro-orchidism.

## Treatment

The patient underwent endoscopic, endonasal trans-sphenoidal hypophysectomy. Safe and effective debulking of the optic chiasm was achieved, with immediate improvement in vision in his right eye following surgery. Histological and immunohistochemical examinations confirmed a pituitary adenoma with a low proliferation index (MIB-1 staining of Ki-67 at 1.3%), patchy positivity for FSH and scattered staining for luteinizing hormone (LH), consistent with a locally infiltrative, gonadotroph adenoma. Immunohistochemical staining was negative for prolactin.

**Figure 2.**
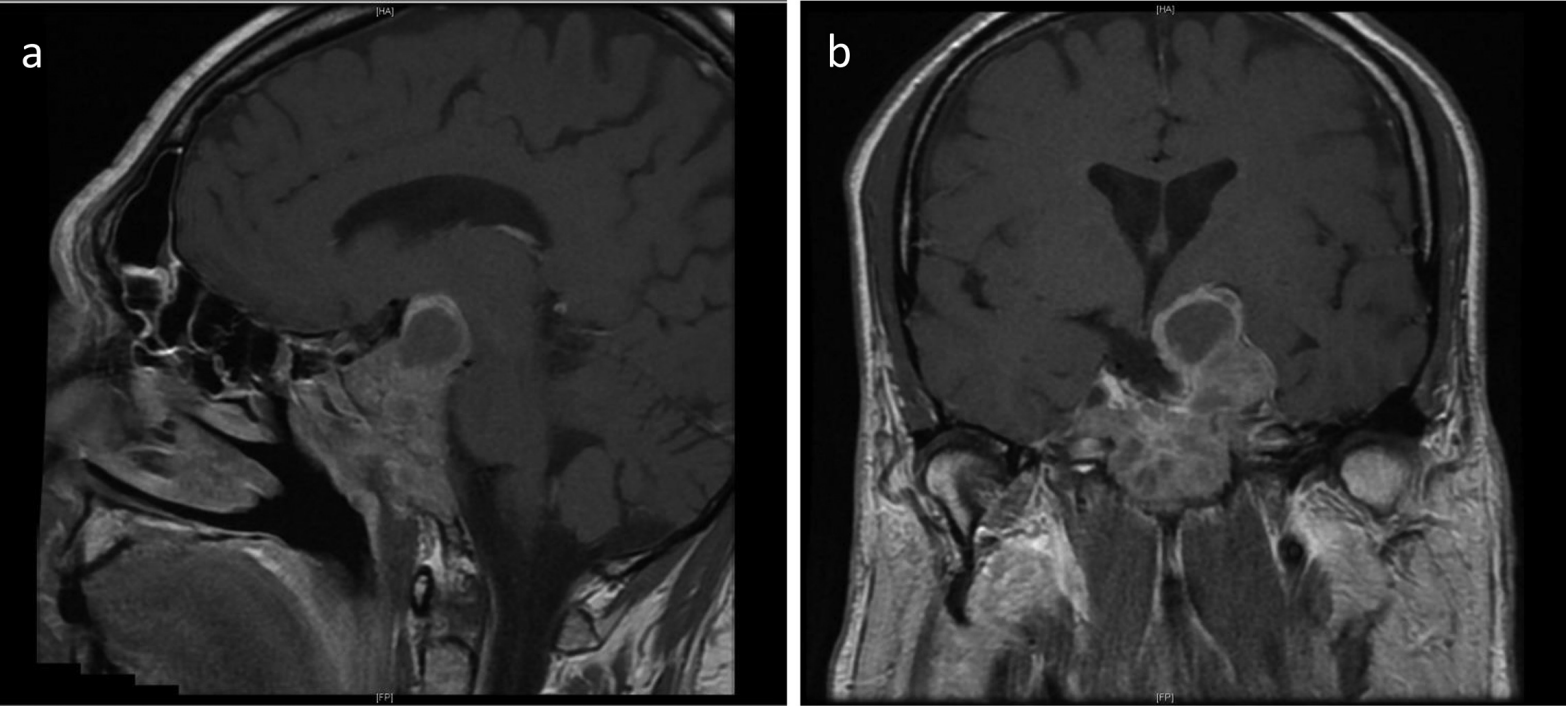
(a) Postoperative *T*_1_ weighted sagittal and (b) coronal MRI scan with contrast demonstrated extensive residual disease at 4 months following decompression surgery. Maximum tumour dimensions were 61, 50 and 34 mm in the craniocaudal, transverse, and anteroposterior planes, respectively.

At 4 months post-operatively, vision in the right eye had returned to normal (visual acuity 6/6, with full resolution of the previous temporal field defect). As anticipated, a repeat MRI scan showed substantial residual tumour, including encasement of the carotid arteries (Figures 2a,b). However, the optic chiasm had been completely decompressed. The patient initially elected for surveillance follow-up, but a further MRI at 9 months post-operatively showed replacement of a cystic, suprasellar component with more solid, enhancing tumour. Additionally, serum FSH remained elevated at 29.6 U l^–1^ (RR 1.0–10.1). Following discussion of the treatment options, including further debulking surgery, the decision was made to proceed with radiotherapy.

**Figure 3.**
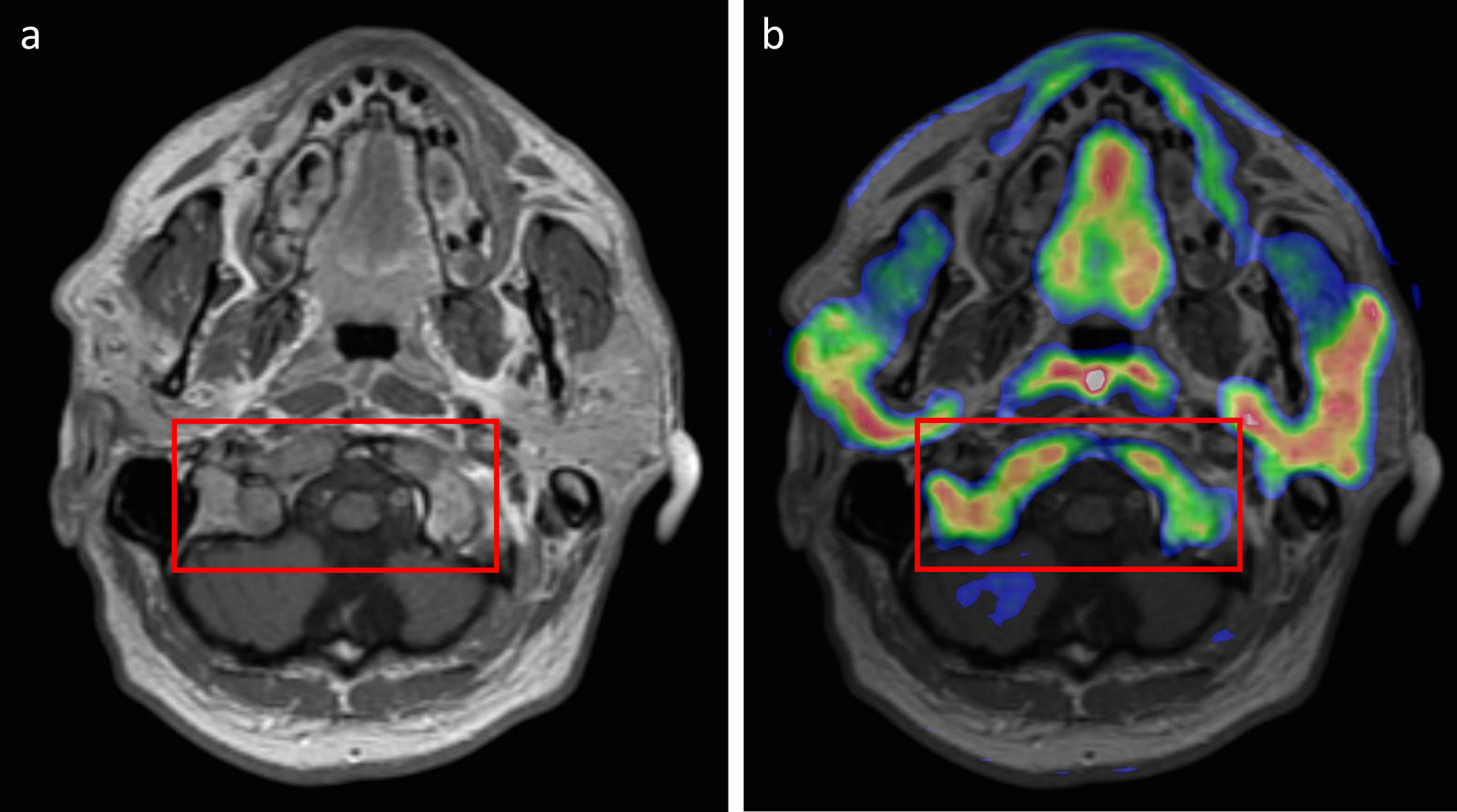
Tumour localisation (indicated by the red box) observed on *T*_1_ weighted axial MRI (a) was confirmed on axial ^11^carbon methionine PET-CT scan (b). Note the normal PET tracer uptake in the parotid glands, seen posterior and lateral to the rami of the mandible, in the posterior wall of the nasopharynx and in the mucosa of the hard palate.

**Figure 4.**
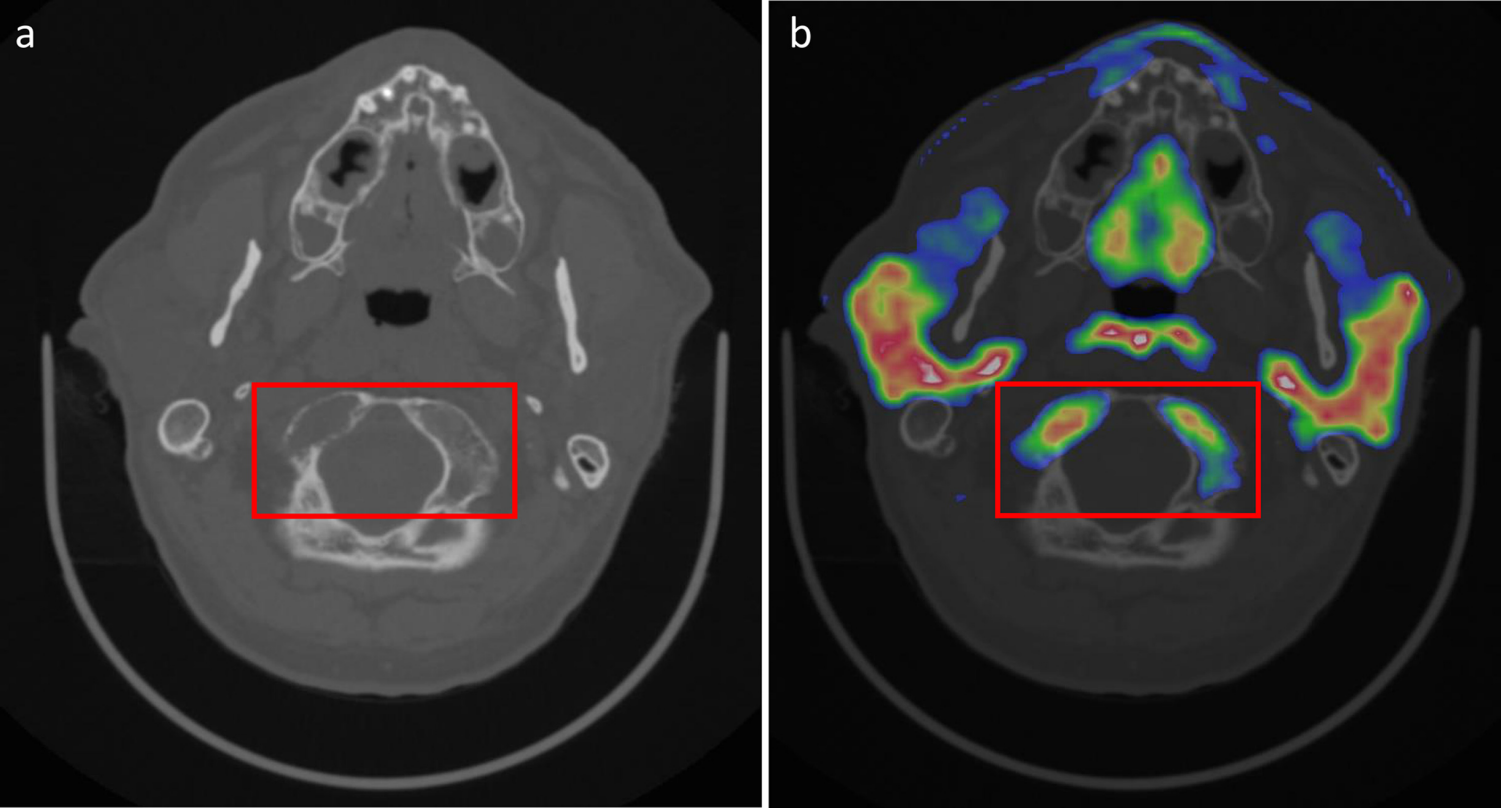
(a) Bony invasion of tumour (indicated by the red box), as evidenced by disrupted trabecular meshwork pattern observed on bone window CT scan, and (b) confirmed on axial ^11^carbon methionine PET-CT scan. Normal PET tracer uptake is seen in the parotid glands, posterior wall of the nasopharynx and the mucosa of the hard palate. Bone marrow uptake in uninvolved bone was not marked.

To perform radiotherapy planning imaging, the patient’s head was immobilized in a thermoplastic shell. A standard planning MRI was obtained, namely a volumetric *T*_1_ weighted sequence with gadolinium contrast, for co-registration to the primary CT scan dataset. Owing to the known bony involvement, a high- resolution bone window CT scan was also performed, and the internal trabecular pattern of the skull was examined to assess the limit of tumour extent. In addition to these scans, a ^11^carbon methionine PET-CT scan was acquired to assist with radiotherapy planning. The patient received an intravenous bolus of 407 MBq of l-[methyl-^11^carbon]-methionine tracer and imaging was performed with a Discovery 690 PET–CT scanner (General Electric Medical Systems, Milwaukee, WI, USA) 20 min following injection. The images confirmed the unusually widespread extent of the residual tumour in the craniocaudal dimension and bony involvement, including inferior invasion into the clivus, pterygoid processes of the sphenoid, basiocciput and right occipital condyle (Figure 3a,b). PET uptake in the bones of the skull base indicated unexpectedly extensive bony infiltration, which, once identified, was confirmed on close and detailed inspection of the bone window CT (Figure 4a,b). While the bulk of the tumour was generally well seen with the combination of MRI and CT, there were areas of uncertainty regarding the true extent of the tumour, which were resolved by the PET imaging. Specific extension of the radiotherapy planning margins to account for areas of uncertainty was not necessitated, thereby minimising the normal tissues included in the target volume. A variable clinical target volume margin of 0.2–0.5 cm was used to reflect the clarity of distinction between the tumour and adjacent structures. Owing to the non-spherical shape of the target, a planning target volume (PTV) margin of 0.5 cm was used, which is larger than our standard PTV margin of 0.3 cm for radiotherapy treatment of pituitary adenomas with daily image guidance. Treatment was delivered with rotational intensity-modulated radiotherapy using TomoTherapy, with additional daily image guidance and positional correction.^4^

**Figure 5.**
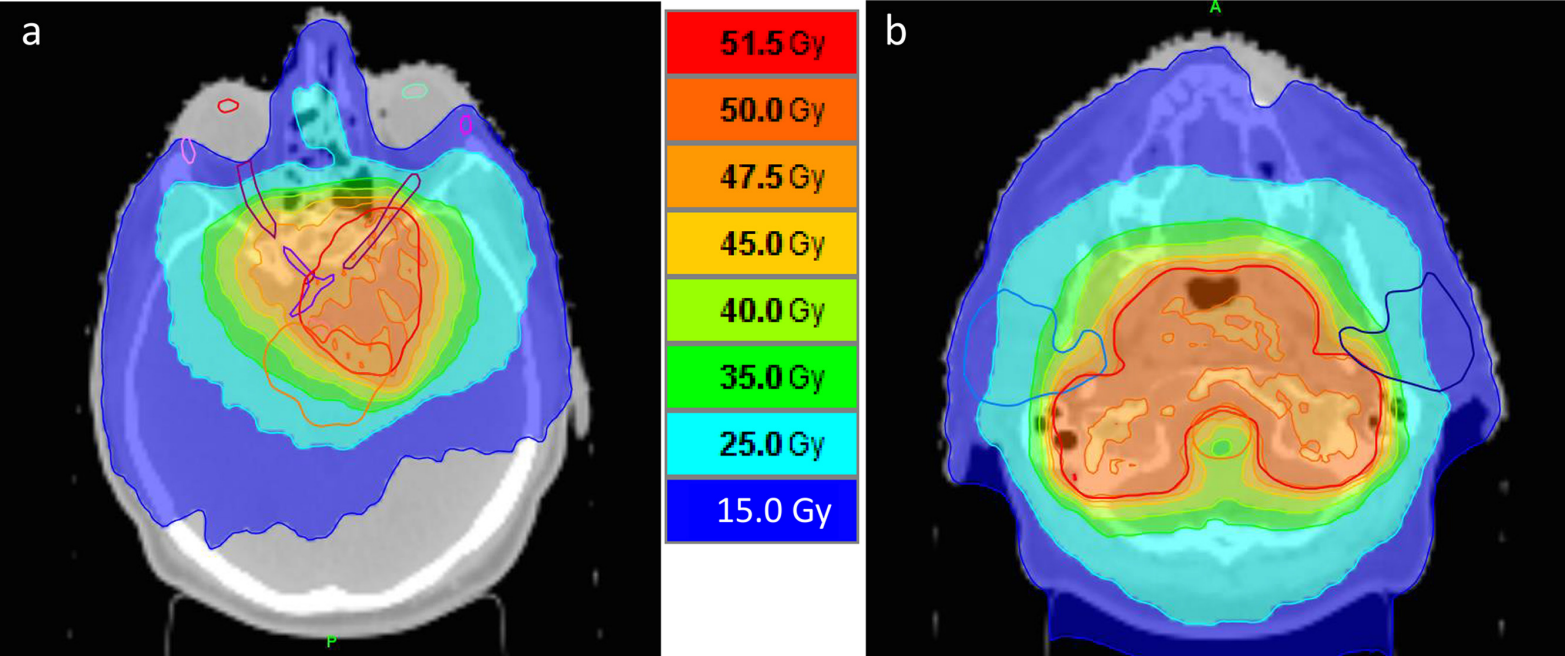
Axial CT scan showing intensity-modulated radiotherapy dose plan. The radiotherapy dose was 50 Gy in 30 fractions over 6 weeks. intensity-modulated radiotherapy was delivered with helical TomoTherapy, using daily image guidance and positional correction.^4^ The planning target volume is shown in red. In (a) the lenses, lacrimal glands, optic nerves and chiasm and brain stem are contoured; in (b) contours for the parotid glands and brain stem are shown. Planning organ at risk volumes for the optic structures and brain stem were created, but are not shown.

The planning objective for the radiotherapy PTV was 50 Gy in 30 fractions to be delivered over 6 weeks (Figure 5). This is a slightly higher dose than is usual in our centre but clinically indicated owing to the size of the residual tumour.^5^ Absolute dose constraints of 50 Gy were set to the planning organ at risk volumes (PRVs) of the brain stem, optic chiasm and optic nerves in order to prevent dose excess. An objective dose for the lenses was set at 4 Gy with an absolute dose constraint at 6 Gy. The left lacrimal gland had an objective dose of 20 Gy with an absolute constraint of 40 Gy, and the right lacrimal gland an objective of 15 Gy with an absolute constraint of 30 Gy. 

Thermoluminescent dosimetry measurements confirmed a predicted total dose to the right lens of 4.23 Gy and the left lens of 4.03 Gy. Mean doses to the left and right lacrimal glands were 20.9 and 14.5 Gy, respectively. For the left and right parotid glands mean doses were 17.9 and 18.5 Gy, respectively, which should be low enough to avoid significant loss of salivary function.^6^ Image-guided intensity-modulated radiotherapy^4^ was completed 16 months following neurosurgical resection.

## Outcome and follow-up

Follow-up is relatively short at present. During radiotherapy the patient reported more acute side effects than are typical for a pituitary adenoma, likely owing to the large tumour volume. Symptoms included sore throat, dry mouth, tinnitus, anorexia and fatigue. The nasopharynx and posterior wall of the oropharynx were included in the PTV. This explains his sore throat, which was treated effectively with parcetamol. His dry mouth resulted from radiation dose received by the parotid glands. His tinnitus may have been related to the dose to the cochleae (average dose of 47 Gy to both). Additionally, although both Eustacian tubes were entirely enclosed within the PTV, he did not complain of symptoms suggestive of secretory otitis media. His fatigue may have been related to brainstem dose.^7^ His FSH level, still elevated at 33.8 U l^–1^ immediately prior to radiotherapy, had reduced slightly to 28.3 U l^–1^ at 6 months after completion of radiotherapy. He currently requires no hormone replacement therapy. The most recent MRI (performed at 21 months post-operatively and 5 months following radiotherapy completion) showed stable disease.

## Discussion

Gonadotroph adenomas constitute 15–40% of pituitary tumours.^8^ Although staining for FSH and/or LH can be clearly demonstrated on immunohistochemistry, the majority are clinically non-functioning and may go undiagnosed until they produce symptoms related to mass effect on adjacent structures. As such, they can grow quite insidiously with invasion of the cavernous sinus and skull base.^9^ Visual deterioration (visual field loss and in more severe cases reduced visual acuity) secondary to compression of the optic chiasm is the most common presenting symptom. In a small number of cases, biologically active FSH (± LH) is secreted, resulting in ovarian hyperstimulation syndrome in females and macro-orchidism in males.^10^ In our patient, serum FSH levels were markedly raised, but there was no clinical or radiological evidence of testicular enlargement.

A pituitary adenoma with maximum diameter of at least 4 cm is subcategorized as a GPA. Neurosurgical intervention is integral to the treatment of GPAs, including the management of tumour-induced visual, endocrine and neurological symptoms.^1^ However, owing to the invasive nature of these tumours, most, if not all (50–100%), patients will have residual disease post-operatively.^1,2^ While a “watch and wait” approach to radiotherapy may be adopted to minimize and delay treatment-induced side effects, adjuvant radiotherapy reduces the likelihood of tumour progression and abnormal hormone secretion in those with significant residual disease.^3^

Standard radiotherapy treatment planning involves the co-registration of MRI, which allows for the appreciation of tumour, to the primary CT data set.^5^ In the case presented here, owing to the large extent of the GPA, its close proximity to several planning organ at risk volumes and the need to administer a larger than normal dose of radiotherapy, the acquisition of additional imaging was judged necessary to appropriately contour the tumour bed. We elected to perform a high-resolution CT scan to detect bony infiltration and a ^11^carbon methionine PET-CT scan to confirm the extent of residual tumour as suspected on both MRI and CT scans. ^11^Carbon methionine PET-CT is a potentially useful adjunct for the imaging of pituitary adenomas, especially when conventional imaging (MRI, CT) is unable to reliably identify the site of primary or recurrent disease or distinguish the latter from post-surgical change.^11,12^ A precursor to protein and polyamine synthesis, methionine is key to cell growth, proliferation and survival. In the central nervous system, ^11^carbon methionine tracer displays preferential uptake by the pituitary gland. Therefore, radiolabelled methionine can be used to distinguish pituitary adenoma tissue from surrounding structures, including normal brain.^11^ Distinctions between tumour and fibrosis, blood collections and/or cysts can also be made.^12^ Many workers have reported its superiority in terms of pituitary imaging compared with the more commonly used tracer FDG^13,14^ and we selected it as the functional imaging modality of choice, on this basis. In addition, methionine PET has been specifically shown to be superior to fludeoxyglucose PET when imaging skull base tumours.^15^ The PET-CT was particularly helpful in emphasizing widespread infiltration of the GPA, distinguishing between tumour and postoperative scarring, and providing reassurance about the tumour limits. In this way, the PET contributed to minimising the volume of normal tissue receiving radiotherapy.

However, the technique has some limitations: physiological ^11^carbon methionine uptake at sites such as lacrimal glands, parotid glands, nasopharynx, bone marrow and normal pituitary gland needs to be considered. This is relevant in our case, as there was suspected bone infiltration by the tumour and PET signal could therefore have represented methionine uptake either in the bone marrow or pituitary tumour. We carefully reviewed bone window CT, MRI and PET images, which were co-registered in the radiotherapy contouring system, in order to differentiate between normal tissues and tumour. Another important limitation of ^11^carbon methionine is its short half-life (20 min), which translates to a need to use the tracer very soon after production, thus limiting its availability to centres that have a cyclotron on-site.

We report a case of a giant gonadotroph adenoma presenting with compression of the optic chiasm and associated with visual loss. Although initial surgical debulking restored the patient’s vision to his premorbid state, significant residual tumour remained. ^11^Carbon methionine PET-CT provided independent corroboration of the extent of the residual tumour, particularly in relation to skull base invasion. It informed radiotherapy planning by helping to ensure complete inclusion of the target volume while concurrently minimising radiation dose to surrounding normal tissues including the parotid glands, lacrimal glands and inner ears.

## Learning points

1. Although benign, giant pituitary adenomas (≥ 4 cm in maximum diameter) may be associated with significant morbidity. Superior extension with compression of the optic chiasm predisposes patients to visual compromise (Figure 1).
2. As in our case, inferolateral extension with invasion of bony structures such as the skull base renders complete surgical resection difficult. Many patients will have residual disease post-operatively (Figure 2).
3. Adjuvant radiotherapy is often given to GPAs to reduce the likelihood of disease progression. Although MRI and CT provide complementary information to facilitate accurate radiotherapy planning, particular challenges exist in cases with extensive tumour invasion or where there is postoperative scar tissue (Figure 5).
4. ^11^Carbon methionine PET-CT provides additional information to help define tumour extent, delineate radiotherapy planning margins and more reliably distinguish post-surgical change from residual tumour. Such confirmation ensures adequate coverage of the target volume while concurrently minimising radiation dose to organs at risk (Figures 3 and 4).

## Acknowledgements

We are grateful to Ms. Kate Burton for help in the acute care of the patient and to the patient for providing written consent. OK, MG and NB are supported by the NIHR Cambridge Biomedical Research Centre.

## Consent

Written informed consent was obtained from the patient for publication of this case report, including accompanying images.

## References

[1] Mortini P, Barzaghi R, Losa M, Boari N, Giovanelli M Surgical treatment of giant pituitary adenomas: strategies and results in a series of 95 consecutive patients. Neurosurgery 2007; 60: 993–1002. 17538372 10.1227/01.NEU.0000255459.14764.BA

[2] Fisher BJ, Gaspar LE, Noone B. Giant pituitary adenomas: role of radiotherapy. Int J Radiat Oncol Biol Phys 1993; 25: 677–81. 8454486 10.1016/0360-3016(93)90015-n

[3] Park P, Chandler WF, Barkan AL, Orrego JJ, Cowan JA, Griffith KA, et al The role of radiation therapy after surgical resection of nonfunctional pituitary macroadenomas. Neurosurgery 2004; 55: 100–6. 15214978 10.1227/01.neu.0000126885.71242.d7

[4] Burnet NG, Adams EJ, Fairfoul J, Tudor GS, Hoole AC, Routsis DS, et al Practical aspects of implementation of helical tomotherapy for intensity-modulated and image-guided radiotherapy. Clin Oncol 2010; 22: 294–312. 10.1016/j.clon.2010.02.003 20303246

[5] Burnet NG, Harris F, Jena R, Burton KE, Jefferies SJ Central nervous system : Hoskin PJ, Radiotherapy in practice – external beam therapy. 2nd edn Oxford, UK: Oxford University Press 2012; 295–341.

[6] Moiseenko V, Wu J, Hovan A, Saleh Z, Apte A, Deasy JO, et al Treatment planning constraints to avoid xerostomia in head-and-neck radiotherapy: an independent test of QUANTEC criteria using a prospectively collected dataset. Int J Radiat Oncol Biol Phys 2012; 82: 1108–14. 21640505 10.1016/j.ijrobp.2011.04.020 PMC3192313

[7] Gulliford SL, Miah AB, Brennan S, McQuaid D, Clark CH, Partridge M, et al Dosimetric explanations of fatigue in head and neck radiotherapy: an analysis from the PARSPORT Phase III trial. Radiother Oncol 2012; 104: 205–12. 22883107 10.1016/j.radonc.2012.07.005

[8] Chaidarun SS, Klibanski A. Gonadotropinomas. Semin Reprod Med 2002; 20: 339–48. 12536357 10.1055/s-2002-36708

[9] Chang CY, Luo CB, Teng MM, Guo WY, Chen SS, Lirng JF, et al Computed tomography and magnetic resonance imaging characteristics of giant pituitary adenomas. J Formos Med Assoc 2000; 99: 833–8. 11155772

[10] Ntali G, Capatina C, Grossman A, Karavitaki N Clinical review: functioning gonadotroph adenomas. J Clin Endocrinol Metab 2014; 99: 4423–33. 25166722 10.1210/jc.2014-2362

[11] Koulouri O, Steuwe A, Gillett D, Hoole AC, Powlson AS, Donnelly NA, et al A role for ^11^C-methionine PET imaging in ACTH-dependent Cushing's syndrome. Eur J Endocrinol 2015; 173: M107–20. 26245763 10.1530/EJE-15-0616

[12] Bergström M, Muhr C, Lundberg PO, Långström B. PET as a tool in the clinical evaluation of pituitary adenomas. J Nucl Med 1991; 32: 610–5. 2013801

[13] Feng Z, He D, Mao Z, Wang Z, Zhu Y, Zhang X, et al Utility of 11C-Methionine and 18F-FDG PET/CT in Patients With Functioning Pituitary Adenomas. Clin Nucl Med 2016; 41: e130–e134. 26646998 10.1097/RLU.0000000000001085

[14] Crippa F, Alessi A, Serafini GL. PET with radiolabeled aminoacid. Q J Nucl Med Mol Imaging 2012; 56: 151–62. 22617237

[15] Tomura N, Mizuno Y, Saginoya T. PET/CT findings for tumors in the base of the skull: comparison of 18 F-FDG with 11 C-methionine. Acta Radiol 2016; 57: 325–32. 25795702 10.1177/0284185115575342

